# Bristle splaying and abrasive potential of different toothbrushes on the enamel and resin composite after brushing simulation

**DOI:** 10.4317/jced.62585

**Published:** 2025-03-01

**Authors:** Gabriel Felipe de Bragança, Juliane Franco Martins, Caroline Garcia Orsi, Ianca Daniele Oliveira de Jesus, Carlos José Soares, Priscilla Barbosa Ferreira Soares

**Affiliations:** 1 Department of Operative Dentistry and Dental Materials, School of Dentistry, Federal University of Uberlândia, Uberlândia, Minas Gerais, Brazil; 2 Department of Periodontology and Implantology, School of Dentistry, Federal University of Uberlândia, Uberlândia, Minas Gerais, Brazil

## Abstract

**Background:**

Correct oral physiotherapy using toothbrushes is essential for mechanically removing biofilms to reduce caries and periodontal disease, but ideal toothbrush parameters have not yet been fully defined. The aim of this study was to evaluate the bristle splaying and abrasive potential of different toothbrushes on enamel and resin composites after simulated brushing for 30, 90, and 180 days.

**Material and Methods:**

Seventy bovine flat tooth crowns containing a circular cavity on the enamel were restored using a resin composite (Forma, Ultradent) and brushed for 30, 90, and 180 days using seven toothbrushes (n = 10): Colorcare (Bianco Oral Care), Delicare (Bianco), Carbon-Magnetherapy (Bianco Oral Care), SlimSoft Advanced (Colgate), Pro Cuidado (Colgate), Curaprox CS5460 (Curaden), and Curaprox CS3960 (Curaden). The enamel and resin composite surface roughness (Ra) were evaluated at baseline and after brushing. The wear index and wear rate were evaluated using macrophotography and scanning electron microscopy (SEM). Data were analyzed using two-way repeated measurement analysis of variance and Kruskal Wallis followed by Tukey’s and Duns tests (α = 0.05).

**Results:**

The Ra of the resin composite significantly increased after 30 (using SlimSoft), 90 (using SlimSoft, Pro Cuidado, and Carbon), and 180 days (using Colgate Pro Cuidado and CS3960; *P*< 0.001). The Ra of enamel increased significantly after 90 days of using Pro Cuidado, and 180 days using Pro Cuidado and CS5460. Carbon exhibited the highest wear index variation.

**Conclusions:**

CS5460, Carbon, and SlimSoft exhibited higher bristle volumes, narrow filament diameters, and higher wear rates and wear indices with lower Ra alterations. The Ra of the resin composite after 180 days was significantly higher for Pro Cuidado and CS3960. Enamel Ra was significantly higher in Pro Cuidado.

** Key words:**Toothbrushing, dental abrasion, composite resins.

## Introduction

Applying the correct oral physiotherapy using toothbrushes is essential for the removal or mechanical disorganization of dental biofilms to reduce caries and periodontal disease (1). Currently, different types of toothbrushes with different sizes, designs, handles, bristles, and head shapes are commercially available (1-3). However, ideal toothbrush parameters have not yet been fully defined (2).

Abrasive wear is defined as the physical loss of mineralized substances from teeth caused by objects other than the teeth (4). Abrasion has a multifactorial origin and involves several factors that directly or indirectly influence the degree of abrasion in the enamel or resin composite (5). Enamel abrasion is influenced by the abrasiveness of the toothpaste; however, this characteristic is modified by the stiffness of the toothbrush bristle (6). Toothbrushes with bristles having more abrasive potential have been associated with a greater ability to cause non-carious cervical lesions (NCCLs) (7,8). When the bristles slide over the interproximal space, they are bent and released with force over the sides of the teeth, increasing the abrasion potential, which can lead to the development of NCCLs from the interproximal to the cervical space of a tooth (9). Additionally, wear caused by brushing can be considered an etiological factor for the initiation of dentin hypersensitivity (10). Soft bristles may contain a higher concentration of hard filaments and contact area with the substrate surface, increasing the amount of toothpaste that moves over the surface, which may reflect greater abrasion caused by the paste (11).

Regarding bristle design, brushes with flat-finish bristles are considered relatively safe, supporting less abrasion than other models (12). The bristle design, configuration and filament stiffness of different toothbrushes can influence abrasion in hard tissue and resin composite restoration (11,12). However, surface abrasion is not yet well established (5,11-12); therefore, the correlation between different toothbrush designs and bristle characteristics with abrasion must be assessed (5,12).

A smooth surface of the resin composite is important for the clinical success of restorations. Increasing the surface roughness of resin composites is associated with greater plaque accumulation, which increases the susceptibility of the tooth to material degradation, caries, and gingival diseases (13). The resin composite abrasion caused by brushing reduces the brightness of the tooth (14); additionally, this effect is independent of the finishing and polishing protocols used (13). The resin composite surface characteristics directly interfere with the esthetics of restorations (15). Toothbrushes with stiff bristles are known to cause more abrasions than those with softer bristles (16,17). Dental substrates or restorative materials with increased surface roughness generally accumulate and retain more plaque than smooth surfaces (18).

This study aimed to evaluate the influence of different types of toothbrushes on bristle wear and enamel and resin composite roughness after simulated brushing. The null hypotheses were as follows: 1) changes in the roughness of the enamel and resin composite are not enhanced by the different toothbrushes; 2) the wear index and wear rate of the different toothbrushes will not vary, and these variables will not be correlated with increasing Ra values of the enamel and resin composite.

## Material and Methods

Seventy bovine incisors of similar shapes and sizes were collected. The teeth underwent prophylaxis, and the roots were removed using a high-speed water-cooled diamond disc (American Burrs, Palhoça, SC, Brazil). The tooth crown was embedded in polystyrene resin (Redelease, São Paulo, SP, Brazil) and the buccal surface was finished and polished using a 600 sandpaper (3M Oral Care, Sumaré, SP, Brazil) to create a flat surface. Circular cavity preparations, 4 mm in diameter and 2 mm in depth, were prepared using a round diamond bur (nº 3053, KG Sorensen, Cotia, São Paulo, Brazil). The restorations were performed via selective enamel etching using 37% phosphoric acid (Condac 37, FGM, Joinville, SC, Brazil) for 30 s, followed by washing with a water/air spray; the excess water was removed using absorbent paper (Sorella, Canoinhas, SC, Brazil). A self-etching adhesive system (Ambar Universal APS; FGM, Joinville, SC, Brazil) was applied to the enamel and dentin in two layers using a microbrush (Cavibrush; FGM). The solvent was evaporated by applying an air jet for 10 s. The adhesive layer was light-cured for 20 s using an LED light-curing unit (Bluephase G2, Ivoclar Vivadent, Schaan, Liechtenstein) at 1400 mW/cm², which was checked using a MARC Resin Calibrator (BlueLight, Halifax, NS, Canada). A resin composite (Forma, Shade A2, Ultradent, South Jordan, UT, USA) was inserted in a single increment and light-cured for 40 s. The restored tooth was finished using 600, 800, 1000 and 1200 sandpapers (3M, Sumaré, SP, Brazil), then polished using a diamond polishing paste of 6, 3, 1, and 0.25 µm with their respective polishing cloths (Arotec, Cotia, SP, Brazil) for 2 min each using a metallographic polisher (Aropol VV, Arotec). After each polishing step, the samples were cleaned ultrasonically (Thornton, Vinhedo, SP, Brazil) in deionized water for 10 min to remove any residues. During this process the specimens were stored in distilled water at 37°C.

A profilometer (SJ-301, Mitutoyo, Kanagawa, Japan) was used to assess the average roughness (Ra, µm) of the enamel and resin composite substrates. Five measurements were performed at different locations on each substrate per specimen, resulting in 100 measurements per group and 700 measurements overall. The Ra value for each specimen is expressed as the mean Ra value of five measurements extracted on each substrate. A cutting length, rate, and length of 0.25 mm, 0.25 mm/s, and 0.8 mm were used, respectively. Measurements were performed at baseline and after 30, 90, and 180 d of toothbrush simulation.

Seven soft or extra/ultra-soft toothbrushes were selected: Colorcare (Bianco Oral Care, Uberlândia, MG, Brazil), Delicare (Bianco Oral Care), Carbon-Magnetherapy (Bianco Oral Care), SlimSoft Advanced (Colgate Palmolive Company, Jaguaré, SP, Brazil), Pro Cuidado (Colgate Palmolive Company), Curaprox CS5460 (Curaden International AG, São Caetano do Sul, SP, Brazil), and Curaprox CS3960 (Curaden International AG). The toothbrushes were blindly assigned to the sequence of brushing and wear analyses by the evaluators.

The brushing simulation was performed using a brushing machine (Odeme Dental Research, Luzerna, SC, Brazil), where the specimens were mounted in a coupled matrix with the flat surface of the enamel and resin composite facing upward. The toothbrush heads of each group were cut using a diamond disk (KG; Sorensen, Barueri, SP, Brazil) adapted to the brushing machine. Toothpaste (Bianco Pro Clinical, Bianco Oral Care) and artificial saliva (ratio of 2 g of toothpaste to 4 ml of saliva) were mixed (19) and placed in the matrix to cover the surface of the specimens. Each group was subjected to three brushing cycles: 30 days, 7.320 cycles; 90 days, 21.960 cycles; and 180 days, 43.820 cycles (19,20). The vertical load was regulated to 200 g on the brush heads, performing linear movements on the surface of the specimens at 25 ± 1°C. Between brushings, the brushing machine was cleaned and the toothpaste mixture was replaced. At each brushing interval, the toothbrushes were washed with distilled water for 2 min before analysis.

To analyze the wear rate and splaying of toothbrush bristles, an evaluation scale was used, which consisted of a score with numbers increasing from zero to three according to the increase in wear (21):

0. Unable to determine the level of use of the toothbrush;

1. The bristles are scattered, but limited to the tufts;

2. Some tufts are scattered and overlap others, with many tangled bristles;

3. Most tufts overlap, and many bristles are curled and bent.

**Figure 1:**
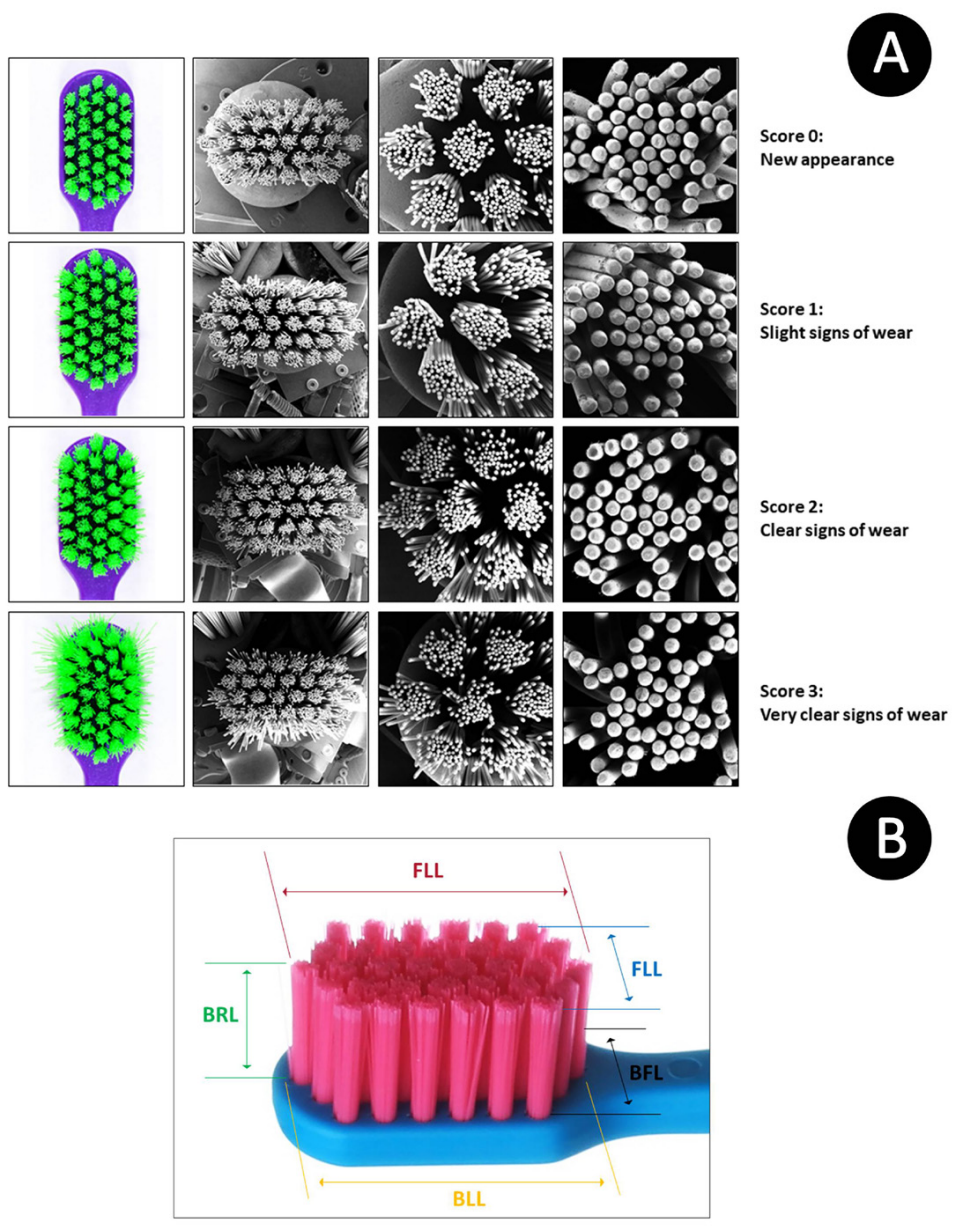
A. Representative image of the wear rate of toothbrushes at the 4 stages of analysis by: Macrophotographs (general aspect of toothbrush), SEM 8 × (general aspect of toothbrush), 25 × (example of one third of the toothbrush head, all 3 thirds were analyzed) and 100 × (image of inner tuft of toothbrush, 6 inner tufts were analyzed from each toothbrush) B. Schematic of the measurement sites for the wear index.

The method consists of visual inspection by three examiners to improve the evaluation and better visualize toothbrush wear. Macrophotography using a DSLR camera with a macro lens and scanning electron microscopy (SEM) at 8 × (one Figure showing all the toothbrushes), 25 × (three images, one per third of the toothbrush), and 100 × (six images showing six inner tufts of the toothbrush) were performed using a representative toothbrush of each group at all four stages of analysis (Fig. 1). The different image acquisition methods (DSLR camera and SEM) and image magnifications were used to compare the methods tested, verify the bristle clutter, and analyze each tuft and deterioration of each bristle at the highest magnification.

**Figure 2:**
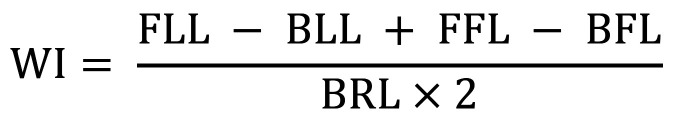
Formula.

To complement the scores from the visual wear rate analysis of toothbrush bristles, the Rawls Wear Index was used, which is based on various bristle measurements and the application of the values in the following formula (21,22): (Fig. 2).

Free long length (FLL) is the extent to which the bristles splay, and is the maximum width of the side of the toothbrush. Base long length (BLL) is the width of the side of the toothbrush at the area fixed to the plastic. Front free length (FFL) is the extent to which the bristles splay, which is the maximum width of the front of the toothbrush. The base-free length (BFL) is the width of the front of the toothbrush at the area fixed to the plastic. The length of the bristles (BRL) is the maximum length of the toothbrush bristles (Fig. 2) (22).

The higher the value obtained, the higher the wear rate. For greater measurement accuracy, images were taken from all angles of the toothbrushes, and measurements were performed using ImageJ software. This process was performed in four stages of analysis.

-Statistical Analysis

Bristle splaying and Ra data were tested for normal distribution (Shapiro–Wilk) and equality of variances (Levene’s test). Two-way analysis of variance (ANOVA) with repeated measurements (RM) was performed for each parameter. Multiple comparisons were performed using the Tukey’s test. The bristle wear data were analyzed using the Kruskal–Wallis and Duns tests. All tests employed α = 0.05 significance level, and all analyses were performed using the statistical package Sigma Plot version 13.1.

## Results

**Figure 3:**
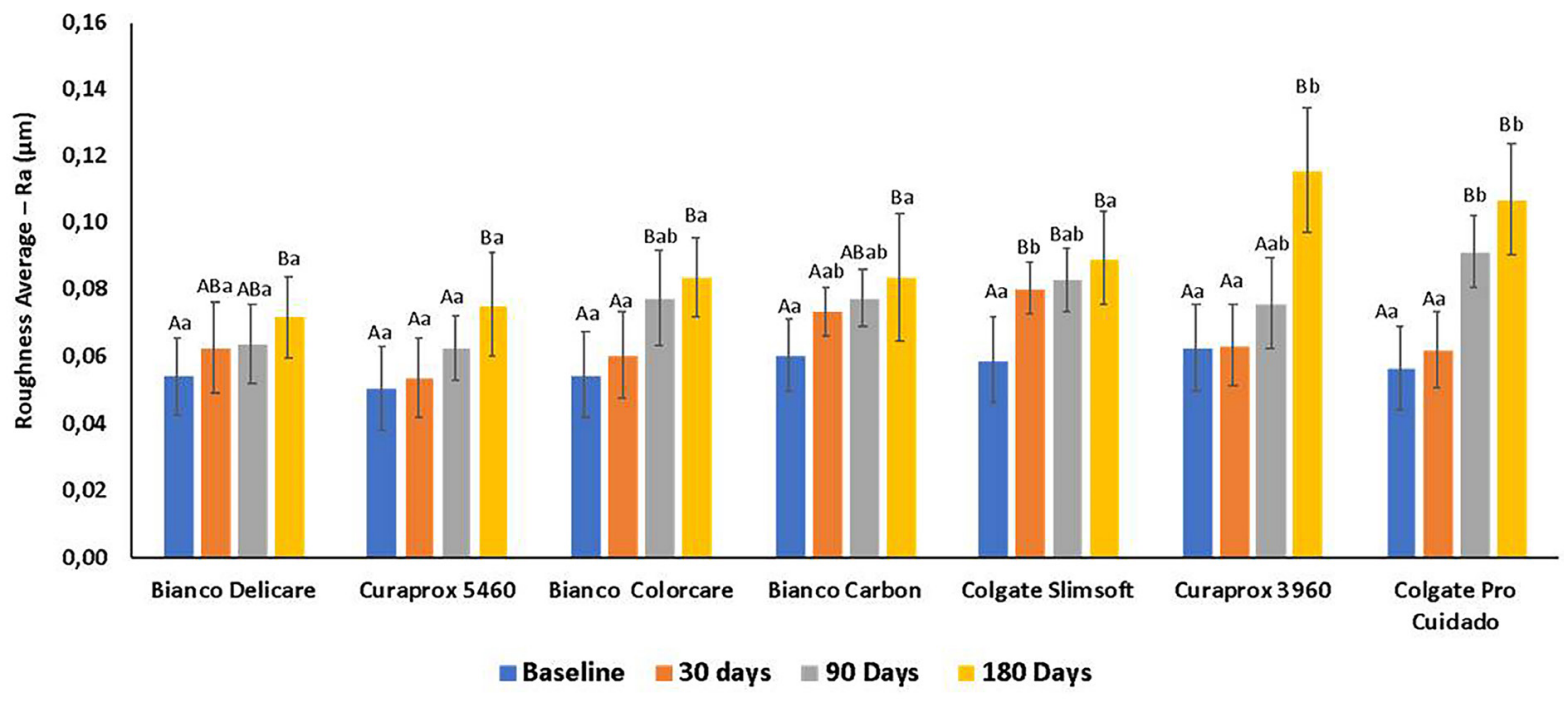
Mean and standard deviation values of the surface roughness (Ra, µm) of the resin composite of all tested toothbrushes at baseline and after simulated brushing for 30, 90 and 180 days. Different letters mean significant difference.

The mean and standard deviation Ra values of the resin composite measured at baseline and after 30, 60, and 90 days of simulated brushing are shown in Figure 3. Toothbrush (*P* < 0.001), brushing time (v < 0.001), and the interaction between toothbrush and brushing time (*P* < 0.001) exhibited significant effects (two-way RM ANOVA). The Ra values were similar at baseline in all groups (*P* = 0.714).

After 30 days of brushing simulation, SlimSoft exhibited significantly higher Ra resin composite values than those of DeliCare, CS5460, Colorcare, CS3960, and Pro Cuidado (*P* < 0.001). After 90 days of brushing simulation, Pro Cuidado exhibited significantly higher Ra resin composite values than those of DeliCare and CS5460 (*P* < 0.001). The Ra values of Pro Cuidado and CS3960 were significantly higher than those of the other toothbrushes (*P* < 0.001) after 180 days of brushing simulation. Compared with the baseline value, the Ra of the resin composite significantly increased after 30 (using SlimSoft; *P* < 0.001), 90 (using SlimSoft, Pro Cuidado, and Carbon; *P* < 0.001), and 180 days (using all toothbrushes).

**Figure 4:**
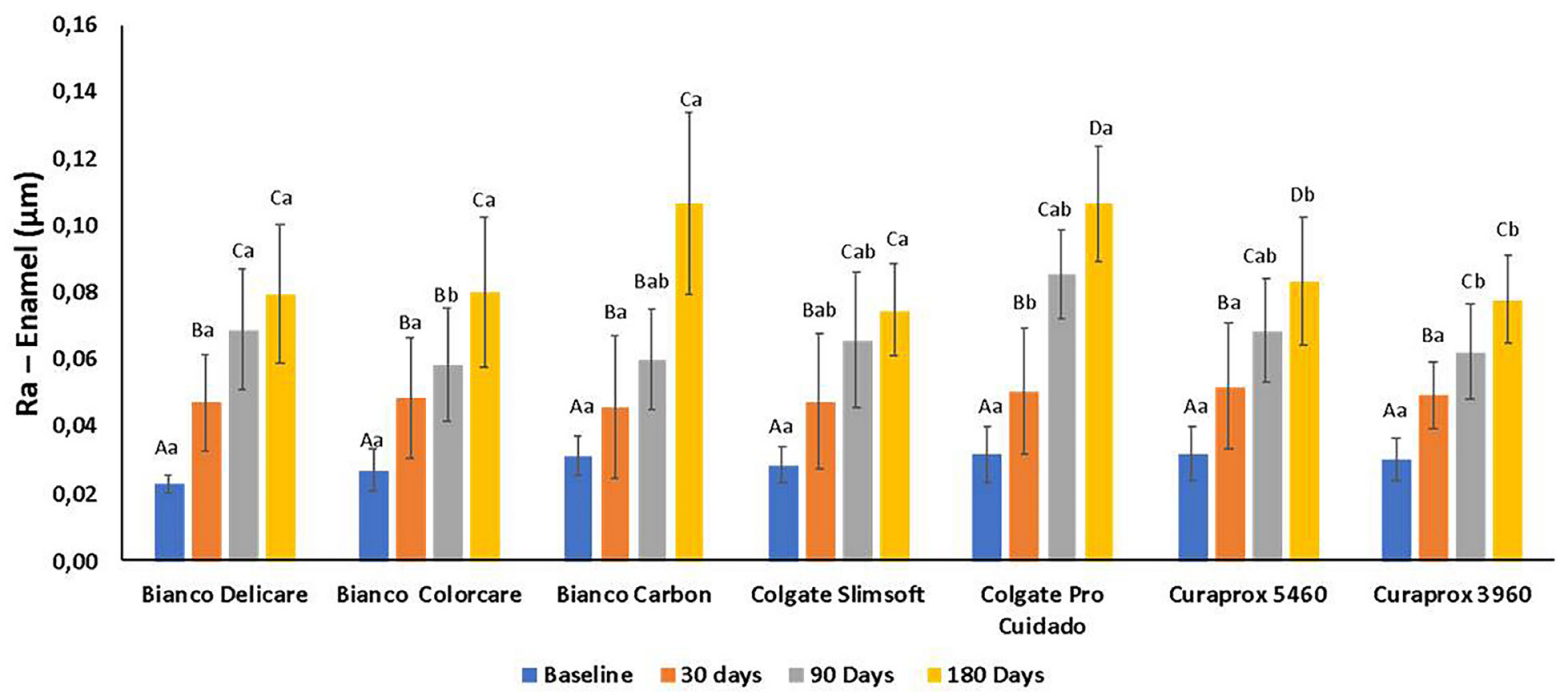
Mean and standard deviation values of the surface roughness (Ra, µm) of the enamel means of all tested toothbrushes at baseline and after simulated brushing for 30, 90 and 180 days.

The mean and standard deviation Ra values of the enamel measured at baseline and after 30, 60, and 90 days of simulated brushing are shown in Figure 4. Toothbrush (*P* < 0.001), brushing time (*P* < 0.001), and the interaction between toothbrush and brushing time (*P* < 0.001) exhibited significant effects (two-way RM ANOVA). The enamel Ra values were similar at baseline in all groups (*P* = 0.714).

After 30 days of brushing simulation, no significant difference was observed between the tested toothbrushes (*P* = 0.398). After 90 days, Pro Cuidado resulted in significantly higher enamel Ra values than those of the other toothbrushes (*P* < 0.001). After 180 days, Pro Cuidado and Carbon exhibited significantly higher enamel Ra values than those of the other toothbrushes (*P*< 0.001). All tested toothbrushes exhibited significantly higher enamel Ra values after 30 days than those at baseline (*P* < 0.001). A significant increase (*P* < 0.001) in the Ra values of Pro Cuidado was observed after 30 and 90 days; however, the Ra values of the other toothbrushes did not significantly vary (*P* = 0.476) over this period. The Ra enamel values measured after 180 days were significantly higher than those measured after 90 days for all toothbrushes (*P* < 0.001).

**Figure 5:**
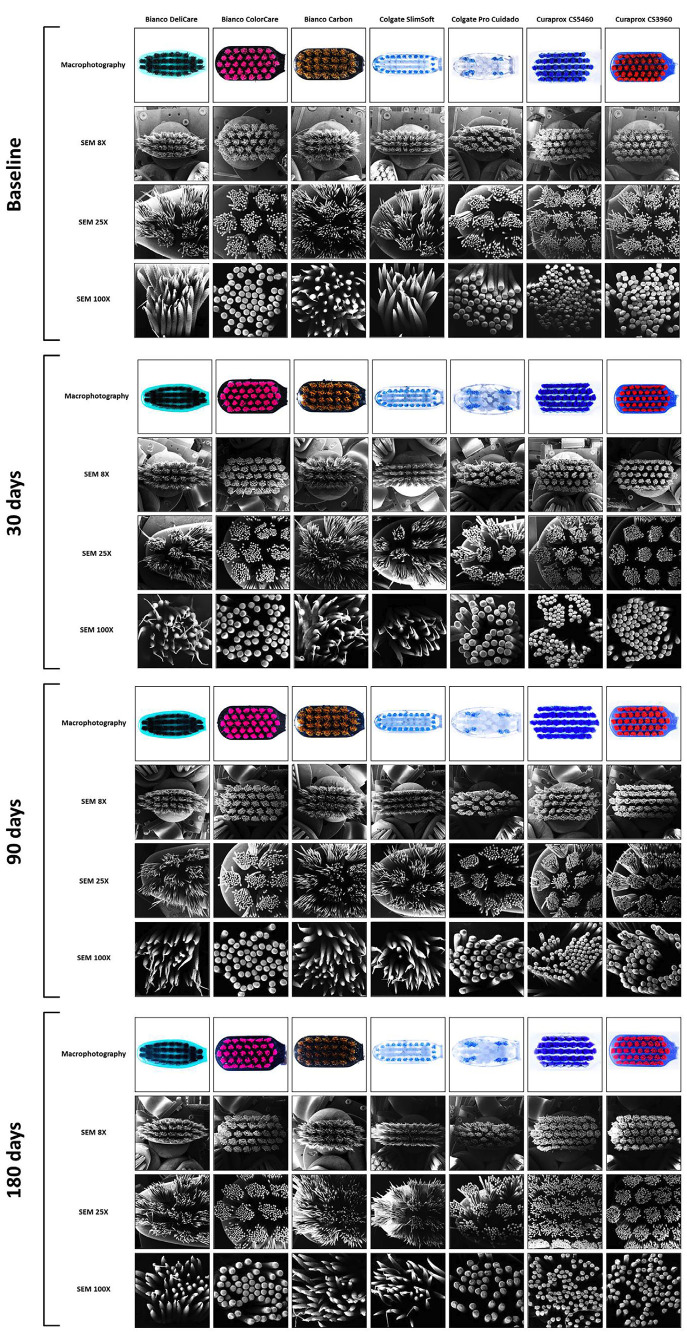
Representative images of the wear rate using macrophotographs and SEM images at 8 ×, 25 × and 100 × at baseline and after 30, 90 and 180 days of brushing simulation.

The means and standard deviations of the toothbrush wear indices are shown in  Table 1. Toothbrush (*P* < 0.001) and time of brushing simulation (*P* < 0.001) exhibited significant effects (two-way RM ANOVA); however, no significant interaction was observed between toothbrush and time of brushing simulation (*P* = 0.121). Figure 5 shows the representative macrophotographs and different SEM magnifications (8 ×, 25 ×, and 100 ×) of the visual wear analysis of each toothbrush; the scores are shown in  Table 2.

Analysis of the macrophotograph showed reduced toothbrush wear and disorganization of the bristles and tufts in all groups, with scores of 0 or 1. SEM analysis using a 8 × magnification (one image showing the entire toothbrush) showed no significant difference in the scores for bristle splaying among the toothbrushes. At a 25 × magnification (three images, each showing a third of the toothbrush) after 90 and 180 days, SlimSoft exhibited significantly higher bristle splaying that of the other toothbrushes. After 180 days of brushing simulation, Delicare and Colorcare frequently scored 1, Carbon, Pro Cuidado, and CS3960 frequently scored 2, and SlimSoft and CS5460 always scored 3. SEM analysis using a 100 × magnification (evaluation of six inner tufts) showed that Bianco Delicare consistently exhibited a score of 2 across all testing periods, whereas that of the other groups exhibited scores of 0 or 1.

## Discussion

This study evaluated the effects of different toothbrushes on the bristle wear rate, wear index, and enamel and resin composite Ra alteration by considering the parameters at baseline and after brushing simulation for 30, 90, and 180 days. Toothbrushes significantly enhanced changes in the Ra of the enamel and resin composite; therefore, the first hypothesis was rejected. Additionally, the toothbrushes exhibited variations in wear index and wear rating; therefore, the second null hypothesis was rejected.

The enamel and resin composite exhibited Ra baseline values of 0.2–0.3 µm and 0.5–0.6 µm, respectively. The nanohybrid resin composite used in this study contained smaller filler particles, was easy to polish, and had gloss-maintenance capacity (23,24). However, the resin composite exhibited higher Ra values than those of the enamel (20). After 30 days, the Ra variation for the enamel was higher than that for the resin composite, regardless of the toothbrush used. A previous study that evaluated the effect of abrasive challenges on the enamel surface and restorative materials by an electric toothbrush and dentifrice used for 2 min, three times a day for 7 days, showed that toothbrush abrasion on enamel was higher than that on restorative materials (25).

A previous study has shown that toothbrushes with a straight-cut bristle design are relatively safer from abrasions (12). However, the bristle cut design was not associated with the Ra variation in the enamel and resin composite of this study. The Pro Cuidado toothbrush exhibited higher enamel and resin composite Ra values than those of the other toothbrushes. The Pro Cuidado toothbrush, which was the only toothbrush composed of spiral brushes, contained a lower volume of bristles than that of the other toothbrushes. This type of bristle may have resulted in the generation of the higher Ra values. The wear index variation between the baseline and 180 days of brushing simulation was higher for the CS5460, Carbon and SlimSoft toothbrushes than that of the other toothbrushes. A direct correlation was observed between ultrasoft toothbrushes that contain numerous bristles and narrow diameters, with the splaying of bristles, and the wear rate of toothbrushes. These toothbrushes likely exhibited a higher visual wear rate in a shorter time than toothbrushes with fewer bristles and larger diameters. A higher toothbrush wear rate resulted in decreased biofilm removal (26). This showed that the toothbrush must be changed when the visual bristles are altered 

This study was limited because the brushing simulation was performed in only one linear direction with a controlled load on the surface of the specimens. Although this methodology has been used in other studies (19,20), it was important to standardize the specimens used in this study. The *in vitro* results must be analyzed with caution because clinical situations may differ due to oral environmental conditions, such as pH, saliva interaction, and pressure variation, which can result in varying toothbrush wear (24). Individuals can use different brushing techniques *in vivo*, such as the application of different pressures/loads, excessive force during brushing, and nonlinear movements (27). Another limitation of this study is the flat enamel-resin composite surface that was prepared to standardize the specimens. Different conditions were examined, including misaligned teeth, irregular surfaces on the anterior and posterior teeth, and metallic and ceramic restorations. The same toothbrush can exhibit different performances when used for patients that use removable dentures with metal clamps, resulting in different toothbrush wear conditions over time.

Despite the use of artificial saliva in combination with toothpaste during simulated brushing, oral conditions, including oral microbiota, pH, and the remineralization effect of natural saliva over time, were not simulated. Natural saliva contains essential substances that promote enamel remineralization, such as calcium, calcium phosphates, polyphosphates, fluoride, and natural products (28). Over the simulated brushing time, no increase in microhardness was observed. Although there is a similarity between artificial and natural saliva in terms of the remineralization effect (29), in simulated brushing, there is not enough time for this effect to occur, which probably influenced the results of Ra in this study.

The formation of NCCLs was attributed to several factors such as the stress concentrated in the cervical region by the occlusal load during mastication and low pH (8,30). However, abrasion/friction caused by brushing has been shown to contribute to the development of such lesions (8,9,30). Patients who brush their teeth with greater pressure tend to exhibit NCCLs (8,9), and high-frequency brushing could be associated with the prevalence of these lesions (30). Additionally, a direct association between bristle hardness and lesion formation has been shown (7). The results of this study confirmed the progressive potential of abrasives for all tested toothbrushes. Careful analysis of the daily use of toothbrushes is recommended, and when visual alterations are verified, a toothbrush change is recommended. When using the same toothbrush, the association between increasing Ra values and biofilm accumulation is clinically difficult to identify; however, this relationship is important. Therefore, after 90 days or when visual bristle splaying has been observed, a change of the toothbrush is recommended.

## Conclusions

The following conclusions were drawn from this *in vitro* study:

Tooth brushes with higher numbers of bristles and narrow filament diameters, such as CS5460, Carbon, and SlimSoft, exhibited a higher wear rate and wear index with lower Ra alterations.

The surface roughness after 180 days was higher with Pro Cuidado and CS3960 on the resin composite, and higher with Pro Cuidado on the enamel.

The wear rate evaluated using SEM magnifications were higher than that from the analysis of the macrophotographs.

After 90 days of simulated brushing, the higher wear rate, bristle splaying, and increase in enamel and resin composite Ra confirmed that the toothbrushes should be changed.

## Figures and Tables

**Table 1:** Mean and standard wear toothbrush indexs at baseline and after 30, 90 and 180 days of brushing simulation.

Toothbrushes	Baseline	30 days	90 days	180 days
Curaprox CS3960	0.05 (0.00) Aa	0.05 (0.01) Aa	0.06 (0.04) Aa	0.09 (0.04) Ab
Curaprox CS5460	0.17 (0.00) Ba	0.21 (0.03) Ba	0.21 (0.05) Ba	0.28 (0.08) Bb
Bianco Colorcare	0.25 (0.00) Ca	0.25 (0.01) Ca	0.27 (0.01) Ca	0.27 (0.01) Ca
Bianco Delicare	0.27 (0.00) Da	0.28 (0.01) Ca	0.29 (0.04) Ca	0.35 (0.04) Cb
Colgate Pro Cuidado	0.28 (0.00) Da	0.30 (0.02) Da	0.30 (0.02) Da	0.35 (0.08) Db
Bianco Carbon	0.34 (0.00) Ea	0.36 (0.04) Ea	0.43 (0.03) Eb	0.45 (0.04) Eb
Colgate SlimSoft	0.32 (0.00) Ea	0.35 (0.01) Ea	0.38 (0.00) Eb	0.40 (0.01) Eb

Different capital letters indicate significant difference of wear index for toothbrush type factor (columns). Different lower-case letters indicate significant different for the moment of the evaluation.

**Table 2:** Evaluation of wear rate for 7 tested toothbrushes at baseline and after 30, 90 and 180 days of brushing simulation, by macrophotographs, SEM 8 x, 25 x and 100 x.

Toothbrushes	Macrophotography				SEM 8x			
	Baseline	30 days	90 days	180 days	Baseline	30 days	90 days	180 days
Bianco Delicare	0	0	0	0	0	1	1	1
Bianco Colorcare	0	1	1	1	0	1	1	1
Bianco Carbon	0	1	1	1	0	1	1	2
Colgate SlimSoft	0	0	0	0	0	0	1	2
Colgate Pro Cuidado	0	0	0	1	0	1	1	2
Curaprox CS5460	0	0	1	2	0	0	2	3
Curaprox CS3960	0	0	0	1	0	0	1	2

## Data Availability

The datasets used and/or analyzed during the current study are available from the corresponding author.

## References

[1] Kalf-Scholte SM, Van der Weijden GA, Bakker E, Slot DE (2018). Plaque removal with triple-headed vs single-headed manual toothbrushes-a systematic review. Int J Dent Hyg.

[2] Jansiriwattana W, Teparat-Burana T (2018). Laboratory Investigation Comparing Plaque Removal Efficacy of Two Novel-Design Toothbrushes with Different Brushing Techniques. Dent J (Basel).

[3] Wu JH, Li JY, Du JK, Lee CY (2023). Comparison of the plaque-removal efficacy of ultra-soft single-headed, triple-headed, and T-shaped toothbrushes and the subjective perceptions of users. J Oral Sci.

[4] Schlueter N, Amaechi BT, Bartlett D, Buzalaf MAR, Carvalho TS, Ganss C (2020). Terminology of Erosive Tooth Wear: Consensus Report of a Workshop Organized by the ORCA and the Cariology Research Group of the IADR. Caries Res.

[5] Dionysopoulos D, Gerasimidou O (2021). Wear of contemporary dental composite resin restorations: a literature review. Restor Dent Endod.

[6] Tanner M, Singh R, Svellenti L, Hamza B, Attin T, Wegehaupt FJ (2023). Effect of Toothbrush Bristle Stiffness and Brushing Force on Cleaning Efficacy. Oral Health Prev Dent.

[7] Turssi CP, Kelly AB, Hara AT (2019). Toothbrush bristle configuration and brushing load: Effect on the development of simulated non-carious cervical lesions. J Dent.

[8] Kitasako Y, Ikeda M, Takagaki T, Burrow MF, Tagami J (2021). The prevalence of non-carious cervical lesions (NCCLs) with or without erosive etiological factors among adults of different ages in Tokyo. Clin Oral Investig.

[9] Nam J, Nguyen DH, Lee S, Heo SM, Park J (2022). Simulation of Non-Carious Cervical Lesions by Computational Toothbrush Model: A Novel Three-Dimensional Discrete Element Method. Sensors (Basel).

[10] Mosquim V, Carneiro GU, Foratori-Junior GA, Honório HM, Gillam DG, Wang L (2023). Knowledge and Attitudes on Preventing and Treating Dentin Hypersensitivity and Its Predicting Factors: A Cross-sectional Study with Brazilian Citizens. Eur J Dent.

[11] Ali AST, Varghese SS, Shenoy RP (2022). Association Between Cervical Abrasion, Oral Hygiene Practices and Buccolingual Dimension of Tooth Surfaces: A Cross-Sectional Study. J Pharm Bioallied Sci.

[12] Bhola L, Unnikrishnan KKR, Chinnannavar SN, Maben S, Sahoo N, Kuruvilla L (2023). In Vitro Assessment of Different Toothbrush Designs on Enamel Surface Abrasion: A Profilometric Study. J Contemp Dent Pract.

[13] Daud A, Adams AJ, Shawkat A, Gray G, Wilson NHF, Lynch CD (2020). Effects of toothbrushing on surface characteristics of microhybrid and nanofilled resin composites following different finishing and polishing procedures. J Dent.

[14] Amaya-Pajares SP, Koi K, Watanabe H, da Costa JB, Ferracane JL (2022). Development and maintenance of surface gloss of dental composites after polishing and brushing: Review of the literature. J Esthet Restor Dent.

[15] AlAli M, Silikas N, Satterthwaite J (2021). The Effects of Toothbrush Wear on the Surface Roughness and Gloss of Resin Composites with Various Types of Matrices. Dent J (Basel).

[16] Souza CMS, Sakae LO, Carneiro PMA, Esteves RA, Scaramucci T (2021). Interplay between different manual toothbrushes and brushing loads on erosive tooth wear. J Dent.

[17] Racki DNO, Comim LD, Dalla Nora Â, Zenkner JEDA, Alves LS (2021). Is Toothbrush Bristle Stiffness Associated with Erosive Tooth Wear in Adolescents? Findings from a Population-Based Cross-Sectional Study. Caries Res.

[18] Yılmaz C, Kanık Ö (2022). Investigation of surface roughness values of various restorative materials after brushing with blue covarine containing whitening toothpaste by two different methods: AFM and profilometer. Microsc Res Tech.

[19] Pinelli LA, Gimenes Olbera AC, Candido LM, Miotto LN, Antonio SG, Fais LM (2017). Effects of whitening dentifrice on yttria-stabilized tetragonal zirconia polycrystal surfaces after simulating brushing. J Prosthet Dent.

[20] Bragança GF, Soares PF, Simeão Borges J, Fernandes Vilela AB, Santos Filho PC, Soares CJ (2022). Effects of Charcoal Toothpaste on the Surface Roughness, Color Stability, and Marginal Staining of Resin Composites. Oper Dent.

[21] Kaneyasu Y, Shigeishi H, Ohta K, Sugiyama M (2022). Analysis of the Deflection, Bristle Splaying, and Abrasion of a Single Tuft of a Polybutylene Terephthalate Toothbrush after Use: A Randomized Controlled Trial. Materials (Basel).

[22] Choi YJ, Lee SB, Jeon CE, Choi JO (207). A study on toothbrush wear index and wear rate in some kindergarten children. Curr Pediatr Res.

[23] Jaramillo-Cartagena R, López-Galeano EJ, Latorre-Correa F, Agudelo-Suárez AA (2021). Effect of Polishing Systems on the Surface Roughness of Nano-Hybrid and Nano-Filling Composite Resins: A Systematic Review. Dent J (Basel).

[24] Pimentel ES, França FMG, Turssi CP, Basting RT, Vieira-Junior WF (2023). Effects of in vitro erosion on surface texture, microhardness, and color stability of resin composite with S-PRG fillers. Clin Oral Investig.

[25] Mulic A, Ruud A, Stenhagen IR, Bruzell E, Tulek A (2023). Deterioration of direct restorative materials under erosive conditions with impact of abrasion and attrition in vitro. Biomater Investig Dent.

[26] Ledder RG, Latimer J, Forbes S, Penney JL, Sreenivasan PK, McBain AJ (2019). Visualization and Quantification of the Oral Hygiene Effects of Brushing, Dentifrice Use, and Brush Wear Using a Tooth Brushing Simulator. Front Public Health.

[27] Kanzow P, Witt C, Lechte C, Barke S, Rohland B, Schmidt A (2024). Effect of different brushing parameters on erosive tooth wear in primary bovine enamel and dentin. PLoS One.

[28] Farooq I, Bugshan A (2020). The role of salivary contents and modern technologies in the remineralization of dental enamel: a narrative review. F1000Res.

[29] Zaharia A, Plescan VG, Atkinson I, Mocioiu OC, Cantaragiu A, Musat V (2017). Remineralization of natural tooth enamel in artificial saliva environment. Revista de Chimie.

[30] Demarco FF, Cademartori MG, Hartwig AD, Lund RG, Azevedo MS, Horta BL (2022). Non-carious cervical lesions (NCCLs) and associated factors: A multilevel analysis in a cohort study in southern Brazil. J Clin Periodontol.

